# Correlation Models between Environmental Factors and Bacterial Resistance to Antimony and Copper

**DOI:** 10.1371/journal.pone.0078533

**Published:** 2013-10-29

**Authors:** Zunji Shi, Zhan Cao, Dong Qin, Wentao Zhu, Qian Wang, Mingshun Li, Gejiao Wang

**Affiliations:** State Key Laboratory of Agricultural Microbiology, College of Life Science and Technology, Huazhong Agricultural University, Wuhan, P.R. China; Belgian Nuclear Research Centre SCK/CEN, Belgium

## Abstract

Antimony (Sb) and copper (Cu) are toxic heavy metals that are associated with a wide variety of minerals. Sb(III)-oxidizing bacteria that convert the toxic Sb(III) to the less toxic Sb(V) are potentially useful for environmental Sb bioremediation. A total of 125 culturable Sb(III)/Cu(II)-resistant bacteria from 11 different types of mining soils were isolated. Four strains identified as *Arthrobacter*, *Acinetobacter* and *Janibacter* exhibited notably high minimum inhibitory concentrations (MICs) for Sb(III) (>10 mM),making them the most highly Sb(III)-resistant bacteria to date. Thirty-six strains were able to oxidize Sb(III), including *Pseudomonas-*, *Comamonas-*, *Acinetobacter-*, *Sphingopyxis-*, *Paracoccus- Aminobacter-, Arthrobacter-, Bacillus-, Janibacter-* and *Variovorax-*like isolates. Canonical correspondence analysis (CCA) revealed that the soil concentrations of Sb and Cu were the most obvious environmental factors affecting the culturable bacterial population structures. Stepwise linear regression was used to create two predictive models for the correlation between soil characteristics and the bacterial Sb(III) or Cu(II) resistance. The concentrations of Sb and Cu in the soil was the significant factors affecting the bacterial Sb(III) resistance, whereas the concentrations of S and P in the soil greatly affected the bacterial Cu(II) resistance. The two stepwise linear regression models that we derived are as follows: 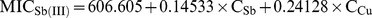
and 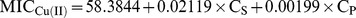 [where the MIC_Sb(III)_ and MIC_Cu(II)_ represent the average bacterial MIC for the metal of each soil (µM), and the C_Sb_, C_Cu_, C_S_ and C_P_ represent concentrations for Sb, Cu, S and P (mg/kg) in soil, respectively, *p*<0.01]. The stepwise linear regression models we developed suggest that metals as well as other soil physicochemical parameters can contribute to bacterial resistance to metals.

## Introduction

Antimony (Sb) and Cu compounds are both considered as hazardous pollutants by the US Environmental Protection Agency (1979) and the Council of the European Communities (1976). The maximum concentrations permissible of Sb and Cu in drinking water have been set by the World Health Organization at 5 µg/L and 10 µg/L, respectively [1]. Sb is a chalcophile element that is commonly found in Cu, coal, Ag and Pd ores [2]. China is the largest producer of Sb and Cu. Over 80% of the world's supply of Sb comes from the Xikuangshan antimony mine located in Hunan province of southwestern China, and the Daye copper mines in the Hubei province produce large quantities of copper (Fig. S1). Sb and Cu contaminations of soil and water are serious concerns in these locations [3]–[4].

Copper (Cu) is an essential element and exists mainly as Cu(I) and Cu(II) in nature [5]. An excess of Cu is toxic and can cause a whole range of health concerns (http://www.ncbi.nlm.nih.gov/pubmed/17454552). In bacteria, Cu may undergo redox changes between Cu(I) and Cu(II), however, the redox change makes copper very dangerous for cells due to the involvement of Fenton-like reactions creating reactive oxygen species (ROS) [6], [7], [8]. Some bacteria have evaluated various Cu detoxification strategies that were mainly mediated by extrusion of Cu(I)/(II) out of the cells and by preventing cellular damage with Cu chaperones [8].

Sb has some chemical and toxicological properties in common with arsenic (As) [9], but Sb resistance in bacteria is much less studied compared to As resistance [10], [11]. The toxicity of Sb is dependent upon its chemical species: elemental Sb is more toxic than its salts, and inorganic species are more toxic than the organic species. Animal studies have shown that Sb trioxide is carcinogenic [2]. In soil and water systems, Sb exists mainly in the antimonite Sb(III) and the Sb(V) forms, and Sb(III) compounds are much more toxic than Sb(V), which is an important consideration for environmental Sb bioremediation [12], [13]. Biotransformation of metals by microorganisms is a useful strategy for bioremediation [14], [15]. Microorganisms can use Sb in oxidation and reduction reactions that play important roles in the biogeochemical cycle of antimony [16]. Heavy metal-resistant bacteria have also been used in bioremediation strategies to adsorb metals, or in combination with plants for phytoremediation of contaminated soil [17].

So far, the knowledge of microbial Sb mechanism is still very limited including the strategies of Sb uptake, efflux, methylation, oxidation and reduction [16]. In *Escherichia coli*, disruption of the glycerol transporter gene *glpF* reduced the uptake amount of Sb(III), clearly demonstrating the role of GlpF in Sb(III) uptake [18]. In *Saccharomyces cerevisiae*, a protein known as Fsp1 that is homologous to GlpF plays a similar role in the uptake of Sb(III). Sb(V) and Sb(III) enter cells by different routes, and the entrance mechanism for Sb(V) remains unknown [19]. Three families of transporters are associated with Sb(III) efflux: the ArsB protein, the Acr3p family and the ABC transporter superfamily [16]. In addition, biomethylation of Sb(III) has been documented for bacteria and fungi, in which the enzyme S-adenosylmethionine methyltransferase appeared to play a pivotal role [20], [21]. To the best of our knowledge, only a small number of Sb(III)-oxidizing bacteria have been reported to date, including *Stibiobacter senarmontii* [22], *Agrobacterium tumefaciens* 5A [23], six strains identified by our group (*Acinetobacter* sp. JL7, *Comamonas* spp. JL25, JL40, and S44, *Stenotrophomonas* sp. JL9 and *Variovorax* sp. JL23) [13], and two strains (*Pseudomonas* sp. S1 and *Stenotrophomonas* sp. A3) identified recently by Hamamura *et al*. [24]. Strain 5A can oxidize both As(III) and Sb(III); however, a strain harboring a mutated version of the As(III) oxidase gene *aioAB*, which is required for the oxidation of As(III), still exhibited Sb(III) oxidation ability [23]. An As(III)-oxidizing *Sinorhizobium* isolate possessing the aerobic arsenite oxidase gene (*aioA*) did not show any Sb(III) oxidation phynotype [24]. The results suggested that bacterial Sb(III) oxidation is catalyzed by a pathway different from the As(III) oxidation pathway catalyzed by Aio [23], [24].

Stepwise linear regression analysis is a statistical technique used to investigate and model the relationships between variables from multi-factor data. This analysis produces an equation that expresses the relationship between a variable of interest and a set of related predictor variables, following a conceptually logical process [25]. It is a method of regressing multiple variables while simultaneously removing unimportant variables. The stepwise linear regression essentially involves performing a standard regression analysis multiple times, each time removing the most weakly correlated variable. In the end, the only remaining variables are those that explain the distribution best [25]. The stepwise linear regression is widely used in plant and food sciences [26], but is less common in microbiological analyses [27].

Most mining areas are heavily polluted by different metals, including Sb and Cu. Therefore, the objectives of this study were to isolate Sb(III)-resistant, Cu(II)-resistant and Sb(III)-oxidizing bacterial strains from soils in different mining areas of China and to analyze the correlations among soil characteristics, bacterial resistance levels and environmental factors. Understanding the correlation between bacterial resistance and environmental factors is important for successful bioremediation of metal contamination.

## Materials and Methods

### Site description and soil sample collection

Eleven soil samples (LS, LH, JC, DF, DC, DN, DS, DA, TF, TM and TC) representing different types of mines (Sb, coal, Cu, Au, Fe and Sn) in China were collected from 2010–2011 (Fig. S1). No specific permissions were required for these location/activities. In addition, the field studies did not involve endangered or protected species.

For each soil sample, one portion was stored at 4°C for bacterial isolation, and another portion was dried and sieved through a 2 mm screen, to determine the main soil properties and total soil antimony content (Table 1). To measure the total Sb and As contents in the soil, we used an approach that combines HPLC with hydride-generation atomic fluorescence spectroscopy (HPLC-HG-AFS) (Beijing Titan Instruments Co., Ltd., China). The Sb and As contents of the soils were extracted as described in Okkenhaug *et al*. [28], and Johnston and Barnard [29], respectively. The physical and chemical features of the soil samples, including organic matter (O-M), S, N, P, NO_3_^−^, Cu, Fe, and pH, were analyzed as described previously by Liao *et al*. [30].

**Table 1:** Soil characteristics of the 11 different mining soils.

Soil	Soil texture/mine type	pH	O-M (g/kg)	S (g/kg)	N (g/kg)	P (g/kg)	Fe (g/kg)	Cu (mg/kg)	NO_3_^−^ (mg/kg)	As (mg/kg)	Sb (mg/kg)
LS	Sandy loam/Sb soil	7.9±0.03	39.34±0.65	0.27±0.03	1.91±0.06	0.14±0.02	0.55±0.03	5.89±0.11	0.85±0.06	87.23±3.60	475.02±11.07
LH	Sandy loam/Sb mine	7.3±0.17	43.00±0.76	0.09±0.02	0.05±0.01	0.81±0.07	20.62±0.74	47.00±2.86	6.22±0.49	0.11±0.01	36425.25±141.1
JC	Sandy loam/Coal mine	7.2±0.11	303.04±4.49	0.18±0.01	3.82±0.69	0.63±0.01	17.96±0.58	91.67±2.90	48.18±0.27	7.45±1.06	11.84±0.93
DF	Sandy loam/Fe mine	7.4±0.21	14.62±0.19	0.43±0.02	0.39±0.03	1.57±0.02	282.46±0.85	5887.50±21.61	51.29±0.40	2.96±1.28	40.48±2.19
DC	Sandy loam/Cu mine	8.1±0.05	18.21±0.10	0.26±0.02	0.11±0.01	0.39±0.05	179.73±1.05	7455.98±25.46	18.65±2.01	4.96±0.09	21.94±3.30
DN	Sandy loam/Sn soil	8.2±0.11	36.98±3.95	0.79±0.03	0.53±0.03	29.27±0.23	79.47±9.67	5183.25±9.17	39.53±0.58	279.44±35.37	89.25±7.81
DS	Sandy/Cu-Fe mine	7.9±0.48	36.57±1.67	4.78±0.27	0.17±0.01	0.34±0.03	17.79±0.21	5072.85±2.73	4.55±0.47	0.42±0.02	4.47±1.85
DA	Sandy/Gold mine	8.4±0.10	12.82±0.55	0.14±0.02	0.30±0.01	1.03±0.03	18.06±3.95	2722.34±4.92	39.53±0.78	5.99±0.64	3.33±0.07
TF	Sandy loam/Fe mine	8.2±0.07	41.82±0.79	0.30±0.02	0.50±0.05	0.01±0.01	30.65±2.79	34.60±1.58	1.41±0.17	6.25±0.08	1.47±0.07
TM	Sandy loam/Mn mine	7.8±0.24	38.61±1.02	0.34±0.02	0.83±0.02	1.50±0.17	29.13±2.22	38.40±0.78	1.41±0.33	32.26±1.77	6.12±0.39
TC	Sandy/Coal mine	7.5±0.04	47.44±6.93	1.60±0.21	2.21±0.21	0.41±0.01	20.68±0.61	42.33±0.92	37.83±2.33	9.15±0.22	5.37±0.06

The LS *etc.* are the soil names shown in Figure S1;pH, 1:2.5 soil-H2O suspension;O–M, organic matter concentration;N: nitrogen concentration;±, Standard deviation (SD) calculated based on triplicates.

### Isolation and identification of Sb(III)/Cu(II)-resistant and Sb(III)-oxidizing bacteria

One hundred gram of soil were supplemented with antimony potassium tartrate (C_8_H_4_K_2_O_12_Sb_2_·3H_2_O) to a final concentration of 10 mg/kg and incubated at 28°C. After one week, 1 g samples of the enriched soils (in triplicate) were added to 9 mL 0.85% sterilized NaCl solution and shaken at 180 r/min for 30 min. The mixture was then serially diluted and plated on chemically defined medium (CDM) plates (For 1 L: MgSO_4_·7H_2_O, 2.0 g; NH_4_Cl, 1.0 g; Na_2_SO_4_, 1.0 g; K_2_HPO_4_, 0.013 g; CaCl_2_·2H_2_O, 0.067 g; Na-lactate, 5.0 g; Fe_2_SO4·7H_2_O, 0.033 g; NaHCO_3_, 0.798 g and agar, 15.0 g; pH 7.2) [31] containing 25 µM C_8_H_4_K_2_O_12_Sb_2_·3H_2_O. The plates were incubated at 28°C for another week. Single colonies were obtained and each Sb(III)-resistant colony was purified repeatedly to obtain pure isolates.

For the Sb(III) oxidation test, each strain was inoculated into 5 mL liquid CDM medium containing 25 µM C_8_H_4_K_2_O_12_Sb_2_·3H_2_O and incubated at 28°C with 180 r/min shaking for 7 d, and the quantity of Sb(V) and Sb(III) was measured using HPLC-HG-AFS [32]. The parameters and conditions for the HPLC-HG-AFS analysis are shown in supplementary material (Table S1). A total of 36 Sb(III)-oxidizing bacteria were found, and 4 strains showing high Sb(III)-oxidizing efficiency were each inoculated into 100 mL liquid CDM medium each (for strains LH3, LH11, DS8 and DA6), incubated at 28°C and shaken at 180 r/min. When the OD_600_ value reached approximately 0.2, the CDM medium was supplemented with 10 µM C_8_H_4_K_2_O_12_Sb_2_·3H_2_O. Every 4 h, 2 mL samples were taken and analyzed for OD_600_ value and the growth of the strains. At the same time, 1 mL cultures were centrifuged, filtered through 0.2 mm filter membranes, and diluted 100 times with sterile ddH_2_O, and the concentration of Sb(III) or Sb(V) was measured using the HPLC-HG-AFS. The known Sb(III)-oxidizing bacterium *A. tumefaciens* 5A [23] was used as a positive control.

Total DNA from each strain was extracted using standard molecular methods. The nearly full-length 16S rDNA was amplified by PCR using the 16S rRNA gene universal primers 27F (5′-AGAGTTTGATCCTGGCTCAG-3′) and 1492R (5′-GGTTACCTTGTTACGACTT-3′) [13]. Colony morphology and 16S rDNA PCR-RFLP were used to eliminate repeated isolates from each sample, as described previously [11].

### DNA sequencing and phylogenetic analysis

DNA sequencing was performed using the ABI 3730XL DNA analyzer from Sunbiotech (Beijing, China). All sequences were compared with the sequences available in NCBI GenBank using a BLASTN search. Multiple alignments were performed using the CLUSTAL_X program [33]. Phylogenetic analysis was performed in PHYML online web server using the maximum-likelihood method [34] with bootstrap analyses based on 1,000 replications. Some reference sequences from the GenBank were used in the construction of the phylogenetic tree for the sake of clarity. The phylogenetic tree was viewed with MEGA 4.0 [35].

### Determination of the bacterial minimal inhibitory concentrations (MICs) for Sb(III), Sb(V) and Cu(II)

The MIC, defined as the lowest concentration of Sb(III), Sb(V) or Cu(II) that inhibited bacterial growth was determined. A portion of each single colony was inoculated into CDM broth containing increasing concentrations of C_8_H_4_K_2_O_12_Sb_2_·3H_2_O, C_12_H_19_Na_3_O_18_Sb_2_·9H_2_O and CuSO_4_·5H_2_O for Sb(III), Sb(V) and Cu(II), respectively, and the cultures were incubated at 28°C for 7 d.

### Statistical analyses

Stepwise linear regression was used to investigate the impact of physicochemical soil characteristics on the average MICs of the Sb(III)/Cu(II)-resistant bacteria from each soil sample, using the analytical software Statistix 8.0 [36]. Stepwise linear regression built a regression model by iteratively including or eliminating items from a list of independent variables. The average MIC of each soil was calculated as “average MIC of each soil  =  the total MICs of all bacteria of each soil/bacterial members of each soil”. All the environmental factors (Table 1) and the bacterial MIC values (Table S2) were used to generate the stepwise linear regression models. Variables whose *p*-values are bigger than 0.05 would be eliminated (not significant), while the variables whose *p*-values are less than 0.05 would be involved in construction of the models. The *p*-value ≤0.01 is considered as extremely significant. In statistics, the variance inflation factor (VIF) quantifies the severity of multicollinearity in an ordinary least squares regression analysis and provides an index that measures how much the variance of an estimated regression coefficient is increased because of collinearity [36]. Canonical correspondence analysis (CCA) was performed to analysis the correlation between the soil characters and the culturable microbial population structures using the Canoco program for Windows 4.5 (Biometris, Wageningen, Netherlands). CCA is an eigenvalue ordination technique designed for analysis of the relationships between multivariate ecological data which integrates regression and ordination techniques [37]. The 16S rDNA-based sequence similarity was selected to differentiate bacterial population structures [38], [39]

### Nucleotide sequence accession numbers

**Figure 1:**
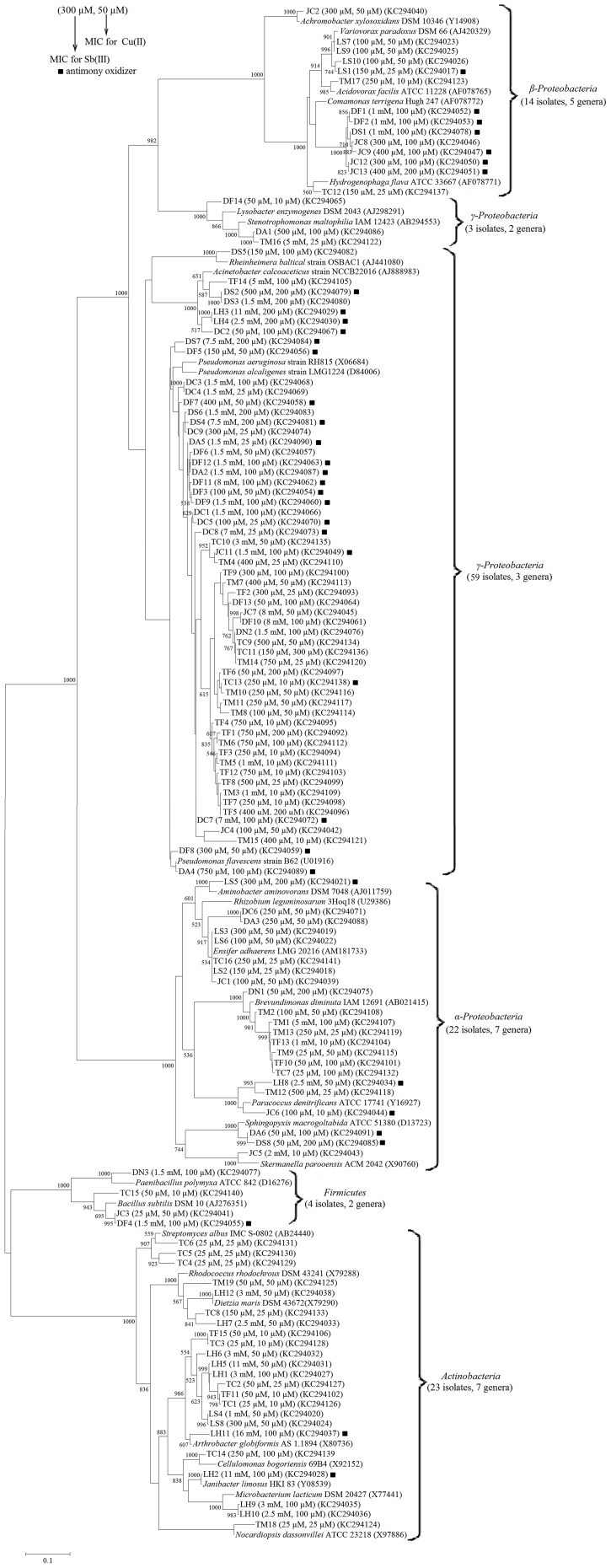
A maximum-likelihood phylogenetic tree on the basis of 16S rRNA gene sequences. The numbers at the nodes indicate bootstrap values (>500) based on 1000 replicates. Bar 0.1, 10 substitutions per 100 nucleotides. The Sb(III)/Cu(II)-resistant bacteria (including Sb(III)-oxidizing bacteria) were isolated using CDM medium as described in Material and Methods. ▪ represents the Sb(III)-oxidizing bacteria. The MIC values for Sb(III) and Cu(II), and the nucleotide accession numbers are shown after each strain's name.

The NCBI GenBank accession numbers for the 16S rRNA gene sequences of the 125 metal-resistant bacteria are shown in Fig. 1.

## Results

### Characterization of soil samples

Eleven mining soil samples representing different types of mines in China were used in this study (supplementary materials, Fig. S1). Several physicochemical soil parameters that potentially influence Sb or Cu metabolism were analyzed. The total Sb concentration was significantly higher in the LH soil (from the Lengshuijiang high Sb content mine) than the other ten soils (LS-TC). The five soils from metal (gold, copper and iron) mines in Daye City (DF, DC, DN, DS and DA) showed significantly higher Cu concentrations compared to the other soil samples. The two coal mine soils (JC and TC) had high organic matter (O–M) and total nitrogen (N) concentrations (Table 1).

### Distribution and diversity of Sb(III)/Cu(II)-resistant bacteria in soils from different mining areas

Analysis of phylogenetic diversity was performed for the bacterial species isolated from the 11 soil samples. Based on colony morphology and 16S rDNA-RFLP analysis, a total of 125 Sb(III)/Cu(II)-resistant bacterial strains were obtained. Overall, 10 (LS1–LS10), 12 (LH1–LH12), 13 (JC1–JC13), 14 (DF1–DF14), 9 (DC1–DC9), 3 (DN1–DN3), 8 (DS1–DS8), 6 (DA1–DA6), 15 (TF1–TF15), 19 (TM1–TM19) and 16 (TC1–TC16) strains were isolated from the LS, LH, JC, DF, DC, DN, DS, DA, TF, TM and TC soils, respectively.

The nearly full-length 16S rDNA sequences of the 125 strains were compared to the GenBank sequences. Fifteen strains showed 100% 16S rDNA sequence identity to at least one available 16S rDNA sequences, 103 strains showed 99%, 4 showed 98% and 3 showed 97% identities (*Paracoccus* spp. JC6, TH8 and TM12). Phylogenetic analysis identified the 125 strains into 27 genera belonging to five major bacterial lineages: *α-Proteobacteria* (22 strains, 7 genera), *β-Proteobacteria* (14 strains, 5 genera), *γ-Proteobacteria* (63 strains, 5 genera), *Actinobacteria* (23 strains, 7 genera) and *Firmicutes* (4 strains, 2 genera) (Fig. 1). Most of the Sb(III)/Cu(II)-resistant isolates were identified as *Pseudomonas* spp. (52/125 = 42%), which were found in all of the soils except for the LS and LH antimony mine soils (Fig. 1; Fig. S2A). Other major Sb(III)/Cu(II)-resistant bacteria were identified as *Arthrobacter* (9%), *Comamonas* (6%), *Brevundimonas* (6%), *Acinetobacter* (5%), *Ensifer* (4%) and *Variovorax* (3%). A number of identified isolates appeared to be specific for a given sampling site: isolates identified as *Aminobacter* and *Variovorax* were only found in LS soil; isolates identified as *Dietzia*, *Janibacter*, and *Microbacterium* were specific for LH soil; isolates identified as *Acidovorax* and *Nocardiopsis* were specific for TM soil; isolates identified as *Achromobacter* and *Skermanella* were specific for JC soil; isolates identified as *Cellulomonas*, *Hydrogenophaga* and *Streptomyces* were specific for TC soil; and isolates identified as *Lysobacter*, *Paenibacillus* and *Rheinheimera* were exclusively found in the Daye area (DF, DN and DS soils) (Fig. 1).

The majority of the Sb(III)/Cu(II)-resistant isolates belonged to the *γ-Proteobacteria* (mainly *Pseudomonas*), *α-Proteobacteria* (mainly *Brevundimonas*) and *Actinobacteria* (mainly *Arthrobacter*) classes. Bacteria from other phylogenetic groups, such as *Firmicutes* and *β*-*Proteobacteria*, were also found, but they were in the minority (Fig. 1; Fig. S2B). *Actinobacteria* was the dominant class in the high Sb-content soil from the LH site. *α-Proteobacteria* and *β*-*Proteobacteria* were the major classes in the LS soil. *γ-Proteobacteria* was the dominant class in the rest of the soils (JC-TC). *γ-Proteobacteria* and *α-Proteobacteria* were found in most of the soil samples. The TC soil sample showed the greatest bacterial diversity, yielding isolates from all five bacterial classes (Fig. S2B).

### The bacterial Sb(III)/Cu(II) resistance levels

Using CDM medium, we determined the MIC for Sb(III) in each of the 125 isolated strains. The MICs ranged from 25 µM to 16 mM (Fig. 1, Table S2). Among the seven dominant genera of the Sb(III)-resistant bacteria, *Acinetobacter* showed the highest average MIC for Sb(III) (3.43 mM, SD = 4.12, n = 6). The other six genera showed different average MIC for Sb(III), in decreasing order from *Arthrobacter* (3.14 mM, SD = 5.37, n = 11) to *Pseudomonas* (1.65 mM, SD = 2.43, n = 52), *Brevundimonas* (0.81 mM, SD = 1.72, n = 8), *Comamonas* (0.63 mM, SD = 0.35, n = 7), *Ensifer* (0.18 mM, SD = 0.09, n = 5) and *Variovorax* (0.11 mM, SD = 0.03, n = 4). In addition, the MIC for Sb(V) was higher than 10 mM in all of the strains, indicating that Sb(V) is much less toxic than Sb(III) for these microorganisms (data not shown).

Certain correlations were found among the Sb(III) resistant levels, the bacterial species and the Sb concentrations of the soils: (1) Among the tested strains, *Arthrobacter* spp. LH11 and LH5, *Acinetobacter* sp. LH3 and *Janibacter* sp. LH2, whose MICs exceeded 10 mM, were all obtained from the LH soil, which was collected from the Lengshuijiang high Sb content mine; (2) The strains isolated from the LH soil had significantly higher average MIC (average MIC = 5.90 mM, SD = 4.86, n = 12) than the other ten soil samples (LS-TC, average MIC = 1.10 mM, SD = 1.95, n = 113); (3) The nine Sb(III)-resistant bacteria with the lowest MIC (25 µM) were all obtained from the three low Sb-content soil samples (TC, TM and JC) (*Arthrobacter* spp. TC1 and TC3, *Streptomyces* spp. TC4, TC5 and TC6, *Brevundimonas* spp. TM9 and TC7, *Nocardiopsis* sp. TM18, and *Bacillus* sp. JC3); (4) The MICs of different strains of the same genus varied greatly. For example, *Arthrobacter* sp. LH11 (16 mM) showed the highest resistance to Sb(III), but *Arthrobacter* spp. TC1 and TC3 (25 µM) showed the lowest MICs. In addition, the MIC of *Brevundimonas* sp. TM1 was 5 mM, but the MIC of *Brevundimonas* sp. TC7 was 25 µM.

The MIC for Cu(II) was also examined in all of the strains using the same method. The MICs for Cu(II) ranged from 10 to 300 µM, which were, in general, much lower than the MICs for Sb(III) (Fig. 1, Table S2).

### Identification of Sb(III)-oxidizing bacteria

**Figure 2:**
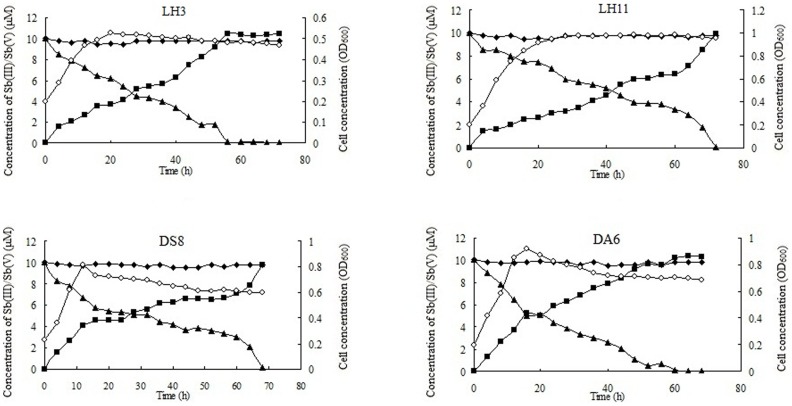
The growth and Sb(III) oxidation curves of the Sb(III)-oxidizing strains identified as *Acinetobacter* sp. LH3, *Arthrobacter* sp. LH11, and *Sphingopyxis* spp. DA6 and DS8. The concentrations of Sb(III) or Sb(V) were analyzed using HPLC-HG-AFS as described in the Material and Methods. ▪, concentration of Sb(V). ▴, concentration of Sb(III). ○, cell concentration (OD_600_). ⧫, concentration of Sb(III) in the controls without bacterial inoculation. The data shown are the representative of three independent experiments.

Out of the 125 Sb(III)/Cu(II)-resistant strains tested, a total of 36 strains showed Sb(III) oxidation ability, including strains identified as 17 *Pseudomonas*, 6 *Comamonas*, 4 *Acinetobacter*, 2 *Sphingopyxis*, and 2 *Paracoccus* strains, and one strain each of *Variovorax*, *Aminobacter*, *Bacillus*, *Janibacter* and *Arthrobacter* (Fig. 1). *Pseudomonas* (47%), *Comamonas* (17%) and *Acinetobacter* (11%) were the 3 major genera and *Pseudomonas* was the most dominant genus of the Sb(III)-oxidizing bacteria. Four of the strains (identified as *Pseudomonas* spp. DF3, DF9 and DF12 and *Sphingopyxis* sp. DA6) showed both Sb(III) and As(III) oxidation. Four strains (identified as *Acinetobacter* sp. LH3, *Arthrobacter* sp. LH11, and *Sphingopyxis* spp. DA6 and DS8) showing high Sb(III) oxidation efficiency were analyzed in detail (Fig. 2). Strain LH3 showed the highest Sb(III) oxidation efficiency (11.9 µM/h•g). The other three strains showed different Sb(III) oxidation rates, decreasing in order from DA6 to DS8 to LH11. No obvious Sb(III) oxidation was observed in the controls without bacterial inoculation (Fig. 2). Among the four strains, LH11 showed the highest resistance to Sb(III) (MIC = 16 mM), but the lowest Sb(III) oxidation rate. Strains DS8 and DA6 showed very low MICs for Sb(III) (both were 50 µM), but higher Sb(III) oxidation rates than LH11 (Fig. 2). There did not appear to be a positive correlation between the Sb(III) oxidation efficiency and Sb(III) resistance level.

### Correlations between the culturable bacterial population structure and the environmental factors

**Figure 3:**
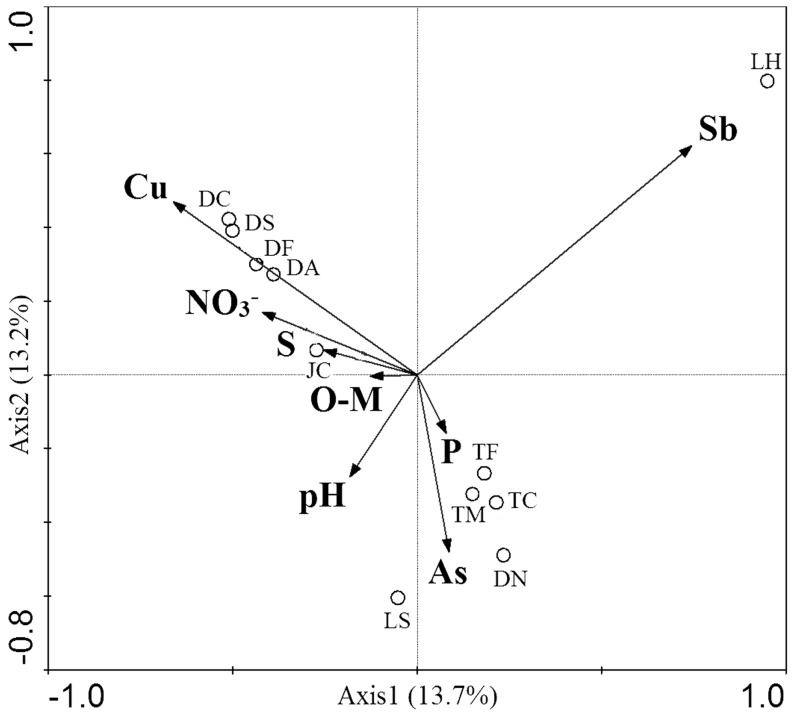
Canonical correspondence analysis (CCA) showing correlations between the culturable microbial population structure and the environmental factors for the 11 soil samples (○, the soil names shown in Fig. S1). The percentages of variation explained by each axis are shown.

The ten environmental variables (Sb, Cu, As, Fe, N, NO_3_^−^, S, P, pH and O-M, Table 1) were analyzed by canonical correspondence analysis (CCA) for correlation with the bacterial population structure. The CCA biplot revealed significant correlations between population structure and eight environmental factors including Sb, Cu, As, NO_3_^−^, S, P, pH and O–M (*p*-value  = 0.03, variable 8; F-ratio  = 1.39; number of permutations  = 999). The total canonical eigenvalue was 6.993. The first canonical axis represented 13.7% of the population structure detected, and the second axis showed 13.2% variance. A total variation of 26.9% was found (Fig. 3). The population structure of the bacteria from the high Sb content LH soil was quite distinct from the other soil samples, and correlated positively with Sb concentration and negatively with pH (Fig. 3). The DF, DC, DS and DA soils clustered together, showing that the four soil samples shared a similar culturable bacterial population structure, which correlated positively with Cu, NO_3_^−^ and S concentrations, demonstrating that the amount of Cu is an important factor affecting the microbial population structure in the four soils. The DN, TF, TM and TC sites clustered together and spread along the second axis, showing a positive correlation with soil As and P concentrations (Fig. 3). The concentrations of Sb and Cu were the two obvious factors affecting the population structure, and the Sb concentration was the most important factor for the microbial population structure.

### Correlation between soil characteristics and the bacterial Sb(III)/Cu(II) resistance levels

In order to determine the impact of the soils' physicochemical characteristics on the bacterial resistances to Sb(III) and Cu(II), we compared the average MIC of the bacteria of every soil against the different environmental factors by stepwise linear regression analysis, since we believe that the average MIC could somehow represent the general bacterial metal resistance level in a sample. Based on the analysis of all the parameters of the 11 soil samples (Table 1), eight environmental factors (O–M, S, N, P, NO_3_^−^, Fe, As and pH) did not reach significant correlation with the average MIC for Sb(III) (*p*>0.05), while the soil Sb and Cu concentrations were significantly correlated with the average MIC for Sb(III) in the bacteria from each soil sample (*p*<0.01) (Table 2). The stepwise linear regression model derived for the 11 soil samples is as follows:
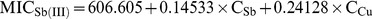
(1)


**Table 2:** Stepwise linear regression models showing the correlations between the bacterial average MIC from each soil and the soil factors (based on the data of the 11 soil samples).

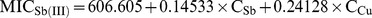 **(R^2^ = 0.9298^a^, *p*<0.01)**				
Variable	Coefficient	Std Error	*P*	VIF^b^
Constant^c^	606.605	184.833	0.0112	1.1
Cu	0.24128	0.04759	0.0010	1.1
Sb	0.14533	0.01273	0.0000	

a R^2^ = 0.9298 or 0.6467 shows that the average MIC was significantly correlated with the soil characters (R^2^>0.362, significant correlation, [32]); ^b^ variance inflation factor; ^c^ intercept. The MIC_Sb(III)_ and MIC_Cu(II)_ are the average bacterial MIC for the metal of each soil (µM), and the C_Sb_, C_Cu_, C_S_ and C_P_ are the soil concentrations for Sb, Cu, S and P (mg/kg), respectively.

Where the MIC_Sb(III),_ C_Sb_ and C_Cu_ represent the average MIC for Sb(III) in the bacteria from each soil (µM), soil Sb concentration (mg/kg) and soil Cu concentration (mg/kg), respectively, *p*<0.01.

Eight environmental factors (O–M, Sb, Cu, N, P, NO_3_^−^, Fe, As and pH) did not reach significant correlation with the average MIC for Cu(II) (*p*>0.05). The average MIC for Cu(II) in the bacteria from each soil was significantly correlated with the soil P and S concentrations (*p*<0.01) (Table 2). The stepwise linear regression model derived for this relationship is as follows:
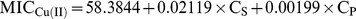
(2)


Where the MIC_Cu(II)_, C_S_ and C_P_ represent the average MIC for Cu(II) in the bacteria from each soil sample (µM), soil S concentration (mg/kg) and soil P concentration (mg/kg), respectively, *p*<0.01.

**Figure 4:**
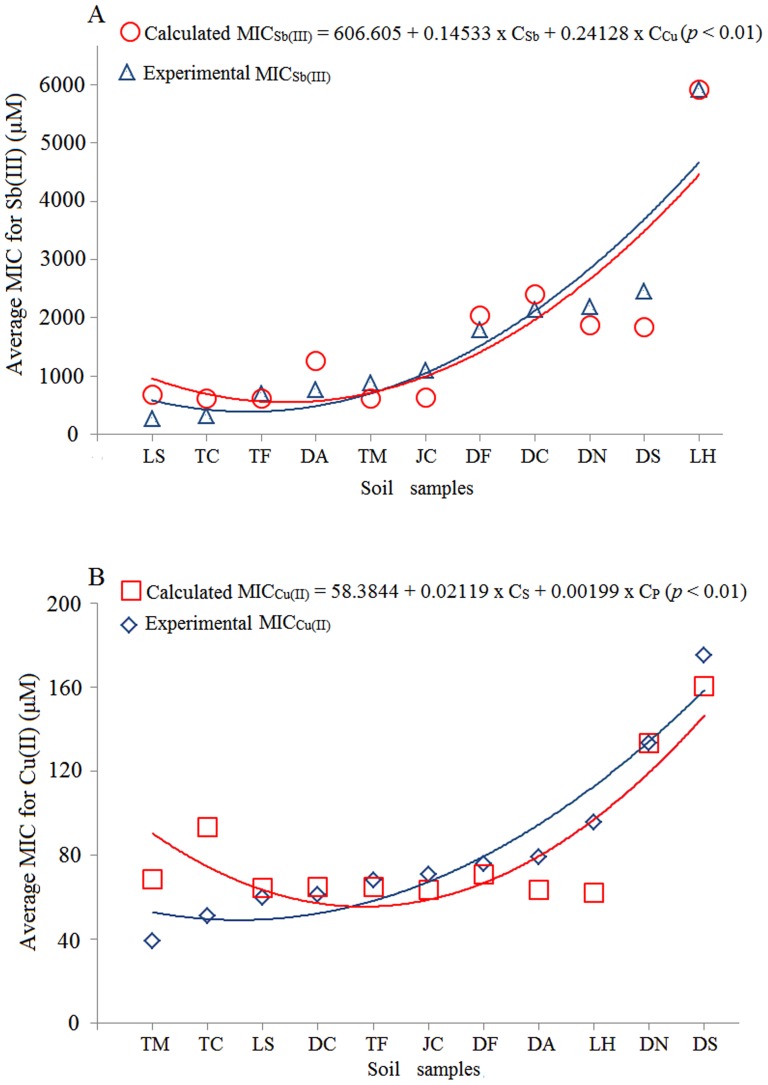
The fitting curves between the experimental MICs and the calculated MICs using the stepwise linear regression models for Sb(III) (A) and for Cu(II) (B). Blue represents the experimental average MIC values tested in the CDM medium. Reds represents the model back-calculated MIC values using stepwise linear regression analysis. The soil names are shown in Fig. S1. The MIC_Sb(III)_ and MIC_Cu(II)_ are the average bacterial MIC for the metal of each soil (µM), and the C_Sb_, C_Cu_, C_S_ and C_P_ are the soil concentrations for Sb, Cu, S and P (mg/kg), respectively.

Fitting curves were used to determine the correlation of the observed average MICs with the back-tested average MICs using the stepwise linear regression models. Based on our analysis of these 11 soils, we showed good correlations and trends between the data using both the experimental values and the back-tested MIC values (Fig. 4, Table 3).

**Table 3:** Comparison between the experimental MIC and the calculated MIC for Sb(III) and Cu(II) in the respective stepwise linear regression models shown in Table 2.

Soil	Range of MICs (mM)	Experimental average MIC for Sb(III) (mM)	Calculated average MIC for Sb(III) (mM)	Absolute value of error	Relative error*(%)	Fitting degree of accuracy ** (%)
LS	0.1–1	0.26	0.68	0.42	61.76	38.24
LH	2.5–16	5.92	5.91	0.01	0.17	99.83
JC	0.025–8	1.10	0.63	0.47	74.60	25.40
DF	0.05–8	1.79	2.03	0.24	11.82	88.18
DC	0.05–7	2.13	2.41	0.28	11.62	88.38
DN	0.05–5	2.18	1.87	0.31	16.58	83.42
DS	0.05–7.5	2.46	1.83	0.63	34.43	65.57
DA	0.05–1.5	0.76	1.26	0.50	39.68	60.32
TF	0.05–5	0.70	0.62	0.08	12.90	87.10
TM	0.025–5	0.87	0.62	0.25	40.32	59.68
TC	0.025–3	0.31	0.62	0.31	50.00	50.00
Average	/	/	/	/	32.17	67.83

Soil	Range of MICs (mM)	Experimental average MIC for Cu(II) (mM)	Calculated average MIC for Cu(II) (mM)	Absolute value of error	Relative error*(%)	Fitting degree of accuracy ** (%)
LS	0.025–0.2	0.06	0.06	0.00	0.00	100.00
LH	0.05–0.2	0.10	0.06	0.04	66.67	33.33
JC	0.01–0.2	0.07	0.06	0.01	16.67	83.33
DF	0.01–0.1	0.08	0.07	0.01	14.29	85.71
DC	0.025–0.1	0.06	0.06	0.00	0.00	100.00
DN	0.1–0.2	0.13	0.13	0.00	0.00	100.00
DS	0.1–0.2	0.18	0.16	0.02	12.50	87.50
DA	0.025–0.1	0.08	0.06	0.02	33.33	66.67
TF	0.01–0.2	0.07	0.06	0.01	16.67	83.33
TM	0.01–0.1	0.04	0.07	0.03	42.86	57.14
TC	0.01–0.3	0.05	0.09	0.04	44.44	55.56
Average	/	/	/	/	22.49	77.51

* Relative error (%) = 100% x (Absolute value of error/ Experimental average);

** Fitting degree of accuracy (%)  = 100% - relative error. The experimental MIC for Sb(III) or Cu(II) of each isolate was tested using the CDM medium as described in the Material and Methods.

### Correlations between the bacterial resistances to Sb(III) and Cu(II)

Since the average MIC for Sb(III) of the Sb(III)-resistant bacteria of each soil were most significantly correlated with the soil Sb and Cu concentrations in the stepwise regression model, we suspected that some correlation may exist between the bacterial resistant level to Sb(III) and Cu(II). Thus, we analyzed the MICs for Sb(III) and Cu(II) of the 125 strains. A unimodal scatter diagram showed that, in general, for each strain, the resistant levels to Sb(III) and to Cu(II) do not appear to be positively correlated, but certain correlation may exist: (1) The strains with low MICs for Sb(III) had a large range MICs for Cu(II) (10 – 300 µM); (2) The strains with high MICs for Sb(III) clustered together and showed MICs for Cu(II) of around 100 µM (Fig. S3).

## Discussion

Microbial oxidation that converts the toxic Sb(III) to the less toxic Sb(V) provides a potential approach for environmental Sb bioremediation [13], [24]. To date, however, there are only a handful of known Sb(III)-oxidizing bacteria, which are strain identified within the genera *Stibiobacter* [22], *Agrobacterium* [23], *Acinetobacter*, *Comamonas*, *Stenotrophomonas*, *Variovorax* [13], *Pseudomonas* and *Stenotrophomonas* [24]. Furthermore, the genes or enzymes required for Sb(III) oxidation are still unknown. Previously, we identified 25 Sb(III)-resistant bacterial strains from Lengshuijiang Sb mined soil which was sampled in 2007 (Li *et al*., 2013). In this study, we obtained 125 Sb(III)-resistant bacteria including 36 Sb(III)-oxidizing bacteria from 11 soils representing different mining areas in China and performed a comprehensive analysis to understand the correlation among the soil characteristics, the microbial diversity and the bacterial Sb(III) resistance levels. Interestingly, at this time, we found four strains that show the highest level of Sb(III) resistance in bacteria reported to date (i.e., MICs greater than 10 mM). We also determined several novel Sb(III)-oxidizing bacteria identified as *Aminobacter*, *Arthrobacter*, *Bacillus, Janibacter*, *Paracoccus* and *Sphingopyxis*. Bacteria from the *Agrobacterium*, *Pseudomonas*, *Variovorax* and *Bacillus* genera have been reported to oxidize As(III) [10], [11], [23], [40]; however, members of the *Comamonas* genus appear to oxidize only Sb(III) and not As(III) (Li et al., 2013 and this study). We sequenced the whole genome of the Sb(III)-oxidizing bacterium *Comamonas* sp. S44 and did not find a putative As(III) oxidase gene [13], [41]. In addition, the mutational analysis indicated that another mechanism [i.e. other than the action of As(III) oxidase on Sb(III)] is responsible for Sb(III) oxidation [42]. Recently, we performed bacterial proteomics analysis of Sb(III) and As(III) oxidation and showed significant differences in the induced protein types (manuscript in preparation). Thus, the molecular basis for the oxidation of Sb(III) and As(III) appears to be quite distinct.

Although it is known that the bacteria-mediated oxidation of Sb(III) to Sb(V) is a detoxification process [23], [24], the toxicity of Sb(V) for bacteria has not been determined In this study, we found that the MICs of Sb(V) for the bacterial strains tested were all higher than 10 mM, indicating that Sb(V) is much less toxic than Sb(III). Due to the low solubility of C_12_H_19_Na_3_O_18_Sb_2_·9H_2_O and the precipitates that appeared in the CDM medium when Sb(V) concentration exceeded 10 mM, the upper limit of MICs of Sb(V) was difficult to determine. So far, the entrance mechanism for Sb(V) remains unknown. It is possible that bacterial cells do not take up Sb(V), resulting in Sb(V) 's low toxicity.

The 125 identified Sb(III)-resistant bacterial strains also showed resistance to Cu(II). Microbial resistance levels to different metals are not well characterized. Based on our experience working with different metal(loid)s, it appears that bacteria are more resistant to As(III) than to Sb(III) but much less resistant to Cu(II) compared with other metal(loid)s, The dominant genera within the Sb(III)/Cu(II)-resistant bacterial strains isolated during this study were identified as *Pseudomonas*, *Arthrobacter*, *Comamonas*, *Brevundimonas*, *Acinetobacter*, *Ensifer* and *Variovorax*. We previously described Sb(III)-resistant bacteria from three of these genera: *Acinetobacter*, *Pseudomonas* and *Comamonas* [13]. Other studies reported that *Acinetobacter*, *Agrobacterium*, *Bacillus* and *Pseudomonas* species are commonly found at As-contaminated sites [43]–[47], and indeed, we found three of these species in this study.

One limitation to our study is that the selective medium only isolates culturable bacteria; however, this method has previously been used successfully in microbial ecological research [11], [48], [49]. The major advantage of the culturable approach is the ability to determine the metal resistance and other physiological traits of the strains (e.g., Sb(III) oxidation level). Furthermore, these strains may be very useful for subsequent studies of Sb resistance mechanisms and are important applicable resources for bioremediation purposes. Using the culturable method, we found that the Sb(III) and Cu(II) resistance levels of different strains identified within the same genus differed greatly. Moreover, no positive correlation between the Sb(III) oxidation efficiency and Sb(III) resistance level was found, indicating that Sb(III) oxidation may contribute only partially to Sb(III) resistance; other mechanisms, such as Sb(III) efflux, may also play a role. We have observed that in many strains, the oxidation of As(III) is approximately 100-fold quicker than Sb(III) oxidation (data not shown).

CCA analysis revealed that the culturable bacterial population structure was greatly affected by soil concentrations of Sb and Cu. Our experiments did not show Fe to be a significant driver in population structure. Turpeinen *et al*. [50] reported that microbial community structure and microbial activity are affected by the amounts of As, Cr and Cu in soils near abandoned wood impregnation plants. Xiong *et al*. [51] reported that microbial community structure is affected by the amounts of As, O–M and P in As-contaminated soil. However, other environmental factors, such as spatial isolation, C/N ratio [41] and pH [52], [53], also contribute to community structure in metal-contaminated soils.

Stepwise linear regression models are used in many studies, but their application is less common in the field of microbiology [26]. In this study, for the first time, we created two equation models for the relationship between soil characteristics and the levels of Sb(III) and Cu(II) resistance in culturable bacteria. We also used fitting curves to show the accuracy of our stepwise linear regression models. In rare cases, the relative error was high (Table 3). This may be caused by the inclusion of a small subset of MICs that was much higher or lower than the others. For example, excluding the MIC for strain JC7 (8 mM) from the 13 MICs of the JC soil sample would reduce the relative error from 74.60% to 21.25%; In the LH soil sample, excluding the MICs for strains LH3 and LH4 (each of 200 µM) would reduce the relative error from 66.67% to 25%. In future studies of MICs, outliers may be eliminated to reduce the relative error.

The Stepwise linear regression model (1) indicated that the Sb and Cu concentrations in soil are significant factors affecting the Sb(III) resistance of bacterial strains. Sb is a chalcophile element and commonly found in ores of copper, silver, lead and coal [2]. Sb and Cu are often found in the same environments, which may result in the further evolution of bacterial species that were already well adapted to the high amounts of Sb- and Cu-rich soil. The model (2) showed that the most significant environmental factors affecting the bacterial Cu(II) resistance were the soil concentrations of S and P. Most of the Cu found in nature is in the form of Cu-sulfide, which could explain the importance of S [54]. However, how P concentration affects bacterial resistance to Cu is still not clear. To the best of our knowledge, there is no evidence that Cu and P are metabolized in a similar manner at the molecular level. Further analysis of bacterial resistance to Cu at different P concentrations or gene mutation experiments may shed more light to understand this relationship.

It has been reported that heavy metal concentration is the primary environmental factor favoring the presence of high numbers of heavy metal-resistant bacteria [11], [13]. In this study, we found that strains isolated from soils with high Sb content showed significantly higher Sb(III) resistance than those strains from soils with low Sb content. This is consistent with our previous report that bacterial strains with higher As resistance were isolated from highly As-contaminated soils [11]. However, bacterial resistance levels to Cu(II) did not correlate with soil Cu(II) content. The stepwise linear regression models we developed suggest that metals as well as other soil physicochemical parameters can contribute to bacterial resistance to metals. It is important to be aware of such an association as it may provide useful information on the genetics, physiology, and ecology of microbes in polluted environments and guide the design of in situ bioremediation schemes.

## Supporting Information

Figure S1 **A map showing the location of the 11 soil sampling sites in the P. R. of China.** These sites include: the Lengshuijiang Sb mine (subsurface soil) (LS) (27°45′ N, 111°28′), the Lengshuijiang high Sb content mine (LH) (27°45′ N, 111°28′), the Jixi coal mine (JC) (45°18′ N, 130°57′ E), the Daye iron mine (DF) (30°12′ N, 114°56′ E), the Daye Tonglvshan copper mine (DC) (30°04′ N, 115°01′ E), Daye tin soil (DN) (30°00′ N, 115°01′ E), Daye delafossite with high sulfur content (DS) (29°59′ N, 114°57′ E), the Daye gold mine (DA) (30°03′ N, 114°59′ E), and the Tianjin iron mine (TF), manganese mine (TM) and coal mine (TC) (39°01′N, 117°11′ E).
(PDF)

Figure S2 (A). Distribution of the isolates of the 52 *Pseudomonas* strains among the 11 soil samples (Figure S1). (B). The percentage of the bacterial classes among the 11 soil samples.
(PDF)

Figure S3 **The unimodal scatter diagram determined using Excel program showing the correlation between the MICs for Sb(III) and for Cu(II) of the 125 Sb(III)-resistant bacterial strains.**
(PDF)

Table S1 **Experimental conditions for HPLC-HG-AFS analysis.**
(PDF)

Table S2 **MIC for Sb(III) and Cu(II) (µM) of each strain in the 11 different mining soils.** The soil names are the same shown in Figure S1.
(PDF)
