# Supplementary material for: Correlation Models between Environmental Factors and Bacterial Resistance to Antimony and Copper
Source: PLoS One. 2013 Oct 29;8(10):e78533. doi: 10.1371/journal.pone.0078533 (PMC3812145; doi:10.1371/journal.pone.0078533)
Supplement: Figure S1 — A map showing the location of the 11 soil sampling sites in the P. R. of China. These sites include: the Lengshuijiang Sb mine (subsurface soil) (LS) (27°45′ N, 111°28′), the Lengshuijiang high Sb content mine (LH) (27°45′ N, 111°28′), the Jixi coal mine (JC) (45°18′ N, 130°57′ E), the Daye iron mine (DF) (30°12′ N, 114°56′ E), the Daye Tonglvshan copper mine (DC) (30°04′ N, 115°01′ E), Daye tin soil (DN) (30°00′ N, 115°01′ E), Daye delafossite with high sulfur content (DS) (29°59′ N, 114°57′ E), the Daye gold mine (DA) (30°03′ N, 114°59′ E), and the Tianjin iron mine (TF), manganese mine (TM) and coal mine (TC) (39°01′N, 117°11′ E). (PDF) [file pone.0078533.s001.pdf]

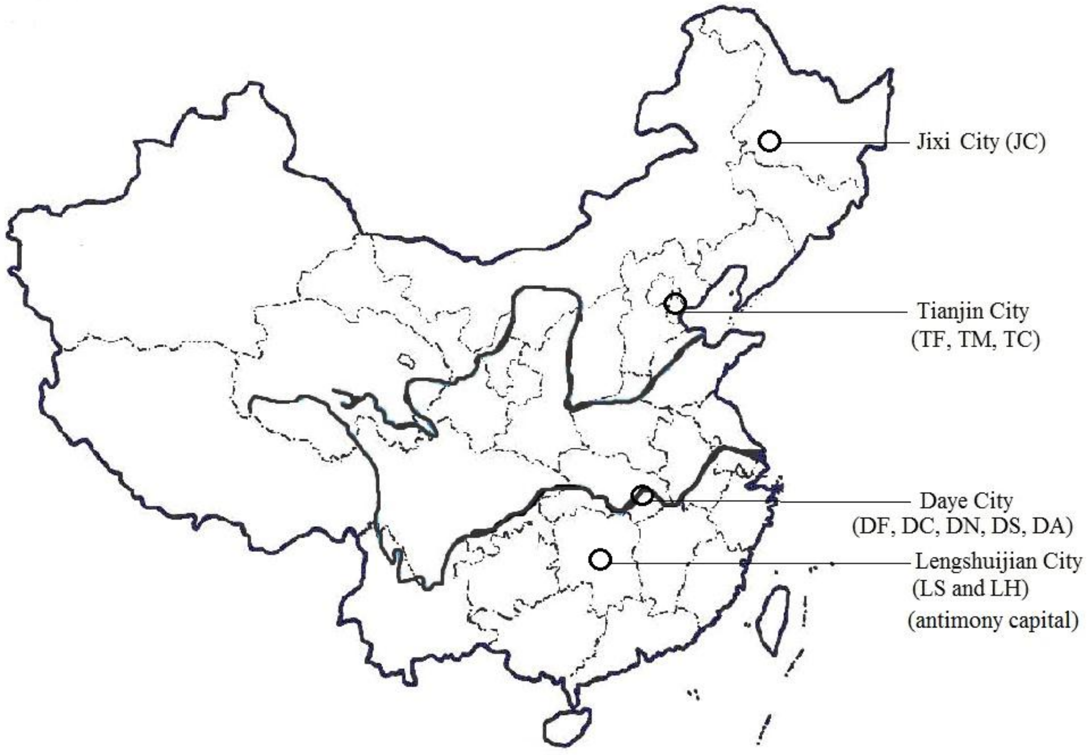

Figure S1

Figure S1. A map showing the location of the 11 soil sampling sites in the P. R. of China. These sites include: the Lengshuijiang Sb mine (subsurface soil) (LS) (27°45' N, 111°28'), the Lengshuijiang high Sb content mine (LH) (27°45' N, 111°28'), the Jixi coal mine (JC) (45°18' N, 130°57' E), the Daye iron mine (DF) (30°12' N, 114°56' E), the Daye Tonglvshan copper mine (DC) (30°04' N, 115°01' E), Daye tin soil (DN) (30°00' N, 115°01' E), Daye delafossite with high sulfur content (DS) (29°59' N, 114°57' E), the Daye gold mine (DA) (30°03' N, 114°59' E), and the Tianjin iron mine (TF), manganese mine (TM) and coal mine (TC) (39°01'N, 117°11' E).
