# Supplementary material for: Correlation Models between Environmental Factors and Bacterial Resistance to Antimony and Copper
Source: PLoS One. 2013 Oct 29;8(10):e78533. doi: 10.1371/journal.pone.0078533 (PMC3812145; doi:10.1371/journal.pone.0078533)
Supplement: Figure S2 — (A). Distribution of the isolates of the 52 Pseudomonas strains among the 11 soil samples (Figure S1). (B). The percentage of the bacterial classes among the 11 soil samples. (PDF) [file pone.0078533.s002.pdf]

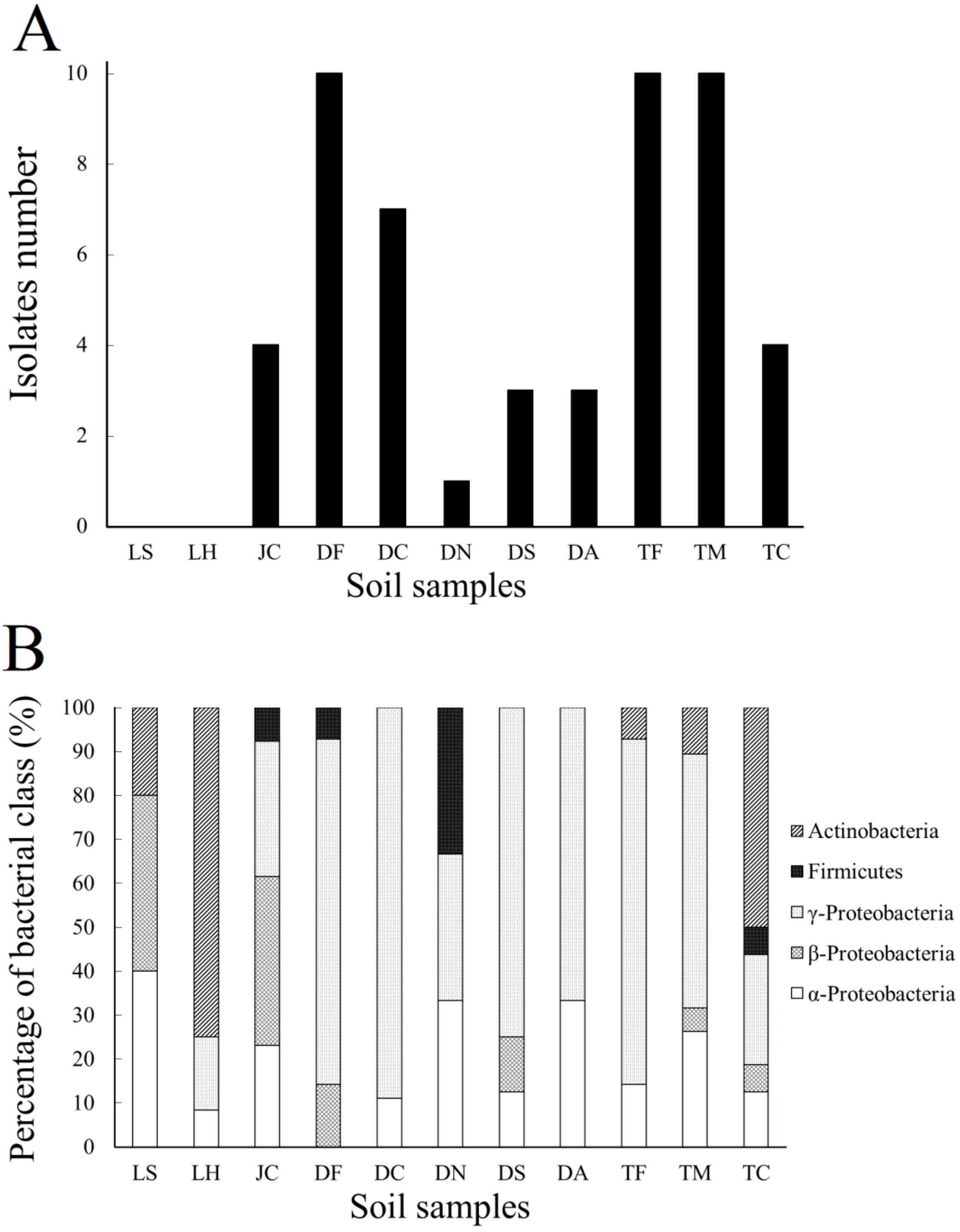

Figure S2

Figure S2 (A). Distribution of the isolats of the 52 *Pseudomonas* strains among the 11 soil samples (Figure S1). (B). The percentage of the bacterial classes among the 11 soil samples.
