# Supplementary material for: Correlation Models between Environmental Factors and Bacterial Resistance to Antimony and Copper
Source: PLoS One. 2013 Oct 29;8(10):e78533. doi: 10.1371/journal.pone.0078533 (PMC3812145; doi:10.1371/journal.pone.0078533)
Supplement: Figure S3 — The unimodal scatter diagram determined using Excel program showing the correlation between the MICs for Sb(III) and for Cu(II) of the 125 Sb(III)-resistant bacterial strains. (PDF) [file pone.0078533.s003.pdf]

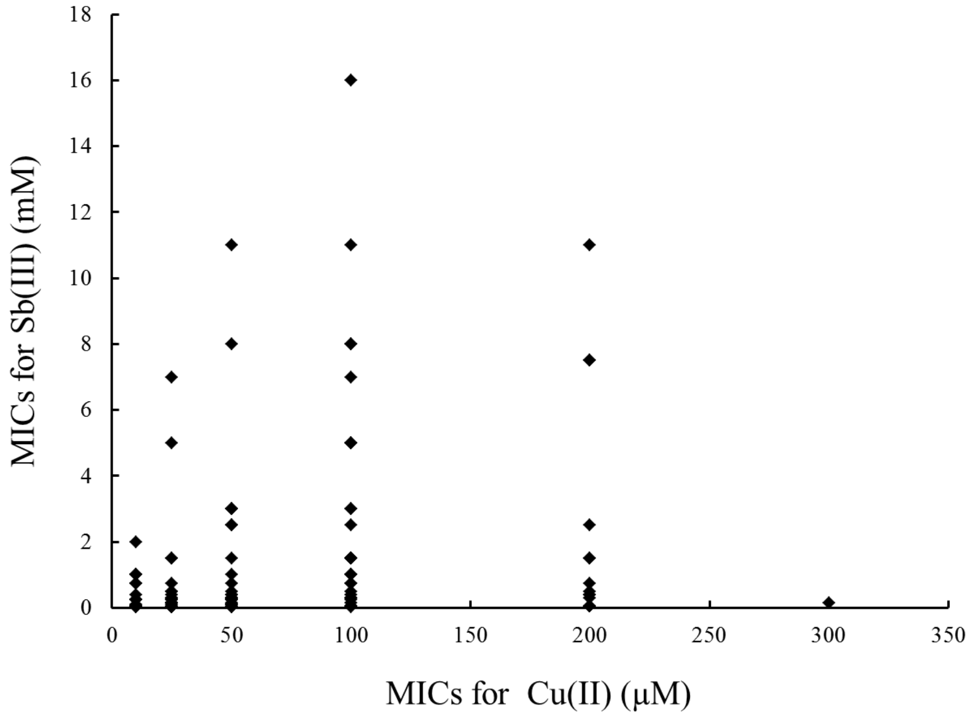

Figure S3

Figure S3. The unimodal scatter diagram determined using Excel program showing the correlation between the MICs for Sb(III) and for Cu(II) of the 125 Sb(III)-resistant bacterial strains.
