# Supplementary material for: Correlation Models between Environmental Factors and Bacterial Resistance to Antimony and Copper
Source: PLoS One. 2013 Oct 29;8(10):e78533. doi: 10.1371/journal.pone.0078533 (PMC3812145; doi:10.1371/journal.pone.0078533)
Supplement: Table S1 — Experimental conditions for HPLC-HG-AFS analysis. (PDF) [file pone.0078533.s004.pdf]

Table S1 Experimental conditions for HPLC-HG-AFS analysis

HPLC	
Column	Hamilton PRP-X100 (250 mm×4.1 mm i.d.,10 μm)
Mobile phase	15 mmol L^{-1} $(NH_4)_2HPO_4$ (pH 6.0)
Flow rate of mobile phase	1.0 mL min^{-1}
Injection volume	100 μL

HG		
KBH_4	1.5% KBH_4 +0.5% KOH	6ml/min
HCl	7%	6ml/min

AFS	
Lamp	Hollow cathode arsenic lamp, 193.7 nm
PMT voltage	280 V
Primary current	90 mA
Carrier gas	Argon, 600 mL min^{-1}
