# Supplementary material for: Correlation Models between Environmental Factors and Bacterial Resistance to Antimony and Copper
Source: PLoS One. 2013 Oct 29;8(10):e78533. doi: 10.1371/journal.pone.0078533 (PMC3812145; doi:10.1371/journal.pone.0078533)
Supplement: Table S2 — MIC for Sb(III) and Cu(II) (µM) of each strain in the 11 different mining soils. The soil names are the same shown in Figure S1. (PDF) [file pone.0078533.s005.pdf]

Table S2 The MIC for Sb(III) and Cu(II) of each bacterial strains isolated from the 11 soil samples*

Strains	Soil	MIC for Sb(III) (μM)	MIC for Cu(II) (μM)	Strains	Soil	MIC for Sb(III) (μM)	MIC for Cu(II) (μM)
LS1	LS	150	25	DS3	DS	1500	200
LS2	LS	150	25	DS4	DS	7500	200
LS3	LS	300	50	DS5	DS	150	100
LS4	LS	1000	50	DS6	DS	1500	200
LS5	LS	300	200	DS7	DS	7500	200
LS6	LS	100	50	DS8	DS	50	200
LS7	LS	100	50	DA1	DA	500	100
LS8	LS	300	50	DA2	DA	1500	100
LS9	LS	100	50	DA3	DA	250	50
LS10	LS	100	50	DA4	DA	750	100
LH1	LH	3000	100	DA5	DA	1500	25
LH2	LH	11000	100	DA6	DA	50	100
LH3	LH	11000	200	TF1	TF	750	200
LH4	LH	2500	200	TF2	TF	300	25
LH5	LH	11000	50	TF3	TF	250	10
LH6	LH	3000	50	TF4	TF	750	10
LH7	LH	2500	50	TF5	TF	400	200
LH8	LH	2500	50	TF6	TF	50	200
LH9	LH	3000	100	TF7	TF	250	10
LH10	LH	2500	100	TF8	TF	500	25
LH11	LH	16000	100	TF9	TF	300	100
LH12	LH	3000	50	TF10	TF	50	100
JC1	JC	100	50	TF11	TF	50	10
JC2	JC	300	50	TF12	TF	750	10
JC3	JC	25	50	TF13	TF	1000	10
JC4	JC	100	50	TF14	TF	5000	100
JC5	JC	2000	10	TF15	TF	50	10
JC6	JC	100	10	TM1	TM	5000	100
JC7	JC	8000	50	TM2	TM	100	50
JC8	JC	300	100	TM3	TM	1000	10
JC9	JC	400	100	TM4	TM	400	25
JC10	JC	750	50	TM5	TM	1000	10
JC11	JC	1500	100	TM6	TM	750	100
JC12	JC	300	100	TM7	TM	400	50
JC13	JC	400	200	TM8	TM	100	50
DF1	DF	1000	100	TM9	TM	25	50
DF2	DF	1000	100	TM10	TM	250	50
DF3	DF	100	50	TM11	TM	250	50
DF4	DF	1500	100	TM12	TM	500	25
DF5	DF	150	50	TM13	TM	250	25

DF6	DF	1500	50	TM14	TM	750	25
DF7	DF	400	50	TM15	TM	400	10
DF8	DF	300	50	TM16	TM	5000	25
DF9	DF	1500	100	TM17	TM	250	10
DF10	DF	8000	100	TM18	TM	25	25
DF11	DF	8000	100	TM19	TM	50	50
DF12	DF	1500	100	TC1	TC	25	10
DF13	DF	50	100	TC2	TC	50	25
DF14	DF	50	10	TC3	TC	25	10
DC1	DC	1500	100	TC4	TC	25	25
DC2	DC	50	100	TC5	TC	25	25
DC3	DC	1500	100	TC6	TC	25	25
DC4	DC	1500	25	TC7	TC	25	100
DC5	DC	100	25	TC8	TC	150	25
DC6	DC	250	50	TC9	TC	500	50
DC7	DC	7000	100	TC10	TC	3000	50
DC8	DC	7000	25	TC11	TC	150	300
DC9	DC	300	25	TC12	TC	150	25
DN1	DN	50	200	TC13	TC	250	10
DN2	DN	1500	100	TC14	TC	250	100
DN3	DN	5000	100	TC15	TC	50	10
DS1	DS	1000	100	TC16	TC	250	25
DS2	DS	500	200				

*The soil names are listed in Fig. S1
